# Birefringence microscopy enables rapid, label-free quantification of myelin debris following induced cortical injury

**DOI:** 10.1117/1.NPh.12.4.045006

**Published:** 2025-10-28

**Authors:** Alexander J. Gray, Rhiannon E. Robinson, Samer A. Berghol, Douglas L. Rosene, Tara L. Moore, Irving J. Bigio

**Affiliations:** a Boston University College of Engineering, Department of Biomedical Engineering, Boston, Massachusetts, United States; b Boston University, Center for Systems Neuroscience, Boston, Massachusetts, United States; c Boston University School of Medicine, Department of Anatomy and Neurobiology, Boston, Massachusetts, United States; d Boston University College of Engineering, Department of Electrical and Computer Engineering, Boston, Massachusetts, United States

**Keywords:** birefringence, myelin, label-free, quantitative, deep-learning object detection, cortical injury

## Abstract

**Significance:**

Myelin breakdown is prevalent in a range of neurodegenerative diseases, aging, and following various forms of trauma. Yet, current imaging techniques have limited capacity for large-scale study of myelin structural damage. A high-throughput, quantitative imaging method would greatly enhance our understanding of myelin degradation in different contexts.

**Aim:**

We aim to establish birefringence microscopy (BRM) as a high-throughput, label-free imaging technique for large-scale, quantitative assessment of myelin pathology in post-mortem brain tissue. BRM has the capacity to provide rapid myelin imaging, which will provide information complementary to other myelin imaging techniques.

**Approach:**

BRM enables label-free structural imaging of myelin with high spatial resolution. We leverage the high-throughput imaging capability of BRM to characterize the distribution of myelin pathology in a rhesus monkey model of cortical injury across the corpus callosum. This framework is applied at two different post-injury survival times (6 and 12 weeks).

**Results:**

We validate BRM for label-free structural imaging of myelin pathology across large regions of tissue (within the corpus callosum) using a fluorescent myelin stain and several immunohistochemical labels. Next, we train and validate a deep learning-based object detection network for automated identification of myelin pathology, using BRM, in the corpus callosum of monkeys with an induced cortical lesion. BRM, paired with deep learning, revealed significantly higher myelin damage through the corpus callosum, resulting from the lesion, in 6-week recovery monkeys compared with 12-week recovery and age-matched controls (P<0.01). There was no significant difference between 12-week recovery monkeys and age-matched controls.

**Conclusions:**

BRM enables large-scale assessment of myelin structural alterations in post-mortem brain tissue. When combined with deep-learning object detection, BRM enables rapid quantification of myelin damage in the corpus callosum after cortical injury. With proper training, this can be extended to study structural changes in other diseases and regions such as Alzheimer’s disease and chronic traumatic encephalopathy as well as normal aging.

## 1 Introduction

In the central nervous system (CNS), myelination is carried out by oligodendrocytes, which wrap extensions of their membrane around axons to create an insulating barrier.^1^^,^^2^ Myelin is a key structure as it provides trophic support to axons and neighboring cells^1^ and enables rapid conduction of action potentials needed for higher order functions such as cognition,^3^^–^^5^ memory,^6^^,^^7^ and motor skills.^1^^,^^8^ Structural alteration and degeneration of myelin has been shown to decrease axonal excitability^9^ and has been correlated with cognitive^10^^–^^13^ and motor impairment.^9^^,^^14^ Demyelinating diseases, such as multiple sclerosis, are prime examples of cases where myelin alteration plays a direct role in functional deficits.^15^^,^^16^ Myelin degradation has also been shown to underlie cognitive and motor impairment in various neurodegenerative diseases, such as Alzheimer’s disease (AD)^11^^,^^17^^–^^21^ and chronic traumatic encephalopathy (CTE)^22^^,^^23^ as well as various forms of trauma (stroke^24^^–^^27^ and traumatic brain injury^28^^,^^29^) and in normal aging.^13^^,^^17^^,^^30^^–^^33^ Nevertheless, the underlying mechanisms, functional progression, and specific roles that myelin degradation plays in these contexts are not fully understood.

There are currently several conventional methods for myelin imaging, but they lack the utility for large-scale assessment. Here, we define large-scale assessment as the ability to image and analyze microscopic structural changes across broad anatomical regions (on the order of several cm^2^), multiple tissue sections, and multiple specimens to better understand the patterns and extent of myelin damage that occur with disease, injury, and normal aging. Electron microscopy (EM) is considered the gold standard for ultrastructural imaging of myelin, providing unparalleled resolution (<1  nm) for imaging the wrapping of myelin lipid bilayers.^10^^,^^34^ However, EM is impractical for large-scale studies due to its limited field of view (FOV) ($<100\;\mu m^{2}$ per image) and time-consuming and complex sample preparation requirements. To combat these limitations, a variety of label-based and label-free optical imaging techniques have emerged to study myelin in the CNS, each with unique advantages and limitations. Common label-based methods include indirect myelin imaging with immunohistochemical (IHC) labeling of myelin proteins such as myelin basic protein (MBP), myelin lipid protein, and myelin-associated glycoprotein (MAG)^35^^,^^36^ or direct myelin labeling with stains such as Luxol Fast Blue (LFB)^37^ and Fluoromyelin.^38^ These methods use exogenous labels that are laborious to apply and introduce variability into imaging and often require the use of detergents or solvents, which result in inadvertent damage to myelin structure.^39^ Moreover, these label-based methods commonly invoke confocal microscopy, which is a point-scanning modality, and is not well suited to imaging large areas or volumes of tissue. In addition to label-based methods, label-free methods such as third-harmonic generation microscopy (THG),^40^ coherent anti-Stokes Raman scattering,^41^^,^^42^ and spectral confocal reflectance (SCoRe) microscopy^43^^–^^46^ offer high specificity and minimal sample preparation for myelin imaging. These approaches, although powerful, are often constrained by cost and system complexity and are also point-scanning modalities in which a laser is raster-scanned to acquire images, limiting their utility in larger-volume studies of myelin degeneration.

Among these techniques, SCoRe microscopy has emerged as a prominent tool for myelin imaging, both *in vivo* and *ex vivo*, to study structurally intact myelin,^43^ for studying myelin changes from demyelination^46^ and for evaluating decompaction models in mice.^44^ SCoRe has been demonstrated for demyelination in a cuprizone mouse model and myelin decompaction;^44^ however, as a point-scanning scheme, it is unclear whether SCoRe can be applied for larger-scale studies in large-brained species or reveal more subtle myelin structural changes. This label-free technique is based on multilayer, thin-film interference-induced backscattering from the multiple interfaces of compacted myelin and its parent axon, allowing for label-free contrast without exogenous labels.^43^ Because SCoRe imaging is in reflectance mode, it can be implemented for imaging myelination *in vivo*^47^ and can also be paired with fluorescence imaging on a laser scanning confocal microscope.^43^^,^^46^ Despite its strengths, SCoRe microscopy has notable limitations. SCoRe enables rapid quantification of myelin content by simple thresholding of the acquired images and looking to determine the percent SCoRe area. However, this does not provide any specific or quantitative structural information on myelin integrity. Moreover, due to the required interferometric effect of SCoRe, it is limited to imaging of longitudinal fibers (parallel to the surface, <10  deg from parallel to the imaging plane),^45^ limiting the utility to study a small subset of brain regions and introducing variability due to limitations on cutting sections with repeatable orientation. These limitations highlight the need for a structural imaging technique to be scalable to larger volume studies of myelin integrity across large brain specimens.

Birefringence microscopy (BRM) is a rapid, label-free widefield (camera-based) optical imaging technique that enables label-free structural imaging of myelin in thin, post-mortem brain sections (<100  μm thick).^39^^,^^48^ BRM is a polarized light imaging (PLI) technique, where contrast is generated by polarization changes induced by birefringent structures, such as the multilayer arrangement of lipids in myelin sheaths.^49^^,^^50^ BRM has been demonstrated for high-resolution structural imaging of myelin in a cortical injury monkey model,^48^ in a mouse model of demyelination by a cuprizone diet,^37^ and for validating fiber trajectories in human brain samples.^51^ Previous BRM studies have investigated factors that provide optimal detection of myelin, for assessing the loss of myelination, or the presence of structurally altered myelin. However, studies that directly quantify myelin damage with BRM and studies correlating BRM measures with functional metrics associated with myelin damage have been limited. BRM has the potential to enable large-scale studies that quantify myelin damage in different contexts, which would enhance our understanding of the relationship between myelin degradation and functional deficits. In addition to BRM, polarization-sensitive optical coherence tomography (PS-OCT) has recently been used to map myelin birefringence in thick, block-face specimens, offering label-free, depth-resolved reconstructions of fiber architecture.^51^^,^^52^ Unlike volumetric PSOCT or widefield 3D-PLI implementations (e.g., Axer et al.), which emphasize mesoscopic tractography and cannot resolve individual myelinated fibers, our optimized BRM pipeline is tuned for sub-micron, diffraction-limited imaging of individual myelinated axons in thin sections.

In this study, we utilize BRM to quantify the extent of myelin structural changes in a rhesus monkey model of circumscribed cortical injury. First, we validate the type of structural damage that occurs in this model using fluorescent labeling with Fluoromyelin and IHC. Next, we train and validate a deep learning-based object detection model for automated identification of structurally altered myelin in the corpus callosum. We use this validated network to quantify the distribution of myelin debris across the corpus callosum, focusing specifically on damage that projects from the lesion site through the corpus callosum (relative to the extent of the induced cortical lesion). Finally, we compare the prevalence of structurally altered myelin between monkeys at different post-injury recovery time points (6 and 12 weeks). Our approach not only provides insights into the spatial distribution of myelin debris in this model but also offers a framework for studying other models of myelin damage, ultimately contributing to a deeper understanding of the relationships between myelin structural integrity and functional and cognitive deficits.

## 2 Materials and Methods

### 2.1 Rhesus Monkey Model of Circumscribed Cortical Injury

Brain tissue used in this study was harvested from 11 adult female rhesus monkeys^53^ aged 16 to 26 years of age (analogous to humans aged 48 to 78 years^54^). Only female monkeys were available at the time the original study was conducted. Monkeys were acquired from World Wide Primates, Inc., or National Primate Research Centers and were maintained in the Animal Science Center of Boston University Medical Campus (fully AAALAC-accredited). Experiments were approved by the Boston University Institutional Animal Care and Use Committee. During the study, monkeys were individually housed in a colony room, and enrichment procedures that met or exceeded USDA requirements were offered. Monkeys were fed commercial monkey chow as well as fruits and vegetables once per day, immediately following testing, and water was available continuously. Before entering the study, monkeys were assessed for pre-existing abnormalities in overall health. As described in Moore et al. (2019),^53^ monkeys were trained to asymptotic performance on a fine motor task of hand dexterity before a surgery to induce a cortical injury targeted to the hand representation of the primary motor cortex (M1). Following surgery, monkeys were randomly assigned to recover for 6 or 12 weeks, during which time monkeys were re-tested on the fine motor task to assess motor impairment and recovery (see Sec. 2.1.1). See Moore et al.,^55^ for details on lesion placement.

#### 2.1.1 Baseline fine motor function

To establish baseline fine motor function of the hand, monkeys were trained on the Hand Dexterity Task (HDT), a modified version of the Klüver board,^53^^,^^55^^,^^56^ for 4 weeks. After pre-training, the dominant hand was determined using free-choice trials.

#### 2.1.2 Lesion of the hand representation of the primary motor cortex (M1)

**Fig. 1:**
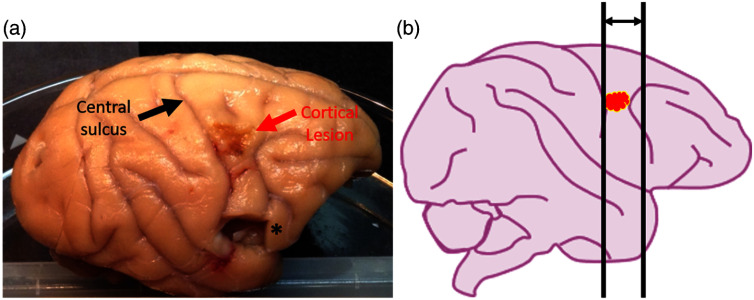
Induction of cortical lesion to the primary motor cortex M1 and section preparation. (a) Photograph of a rhesus monkey brain post-surgery, showing the cortical lesion site in the right hemisphere (red arrow) localized to the primary motor cortex (M1). (b) Schematic illustration of lesion location and coronal sectioning strategy. Coronal sections were collected across the full extent of the lesion and anterior to the lesion, where Wallerian degeneration projects through the corpus callosum (CC). A stereotactic frame was used during brain extraction and sectioning to ensure repeatability and consistency across samples. *Location of tissue biopsy for electrophysical studies.

As described in Moore et al. 2013,^55^ with the monkey fully anesthetized, a craniotomy is performed over the hemisphere contralateral to the dominant hand. The dura is opened, and the precentral gyrus is electrophysiologically mapped using a small silver ball surface electrode to locate the precise area of M1 that controls hand and digit function, delineating the area to receive cortical injury. Using this map, a small incision is made in the pia at the dorsal edge of the hand representation, and a small glass suction pipette is inserted under the pia to bluntly dissect penetrating arterioles from the pia as they enter the underlying cortex in the mapped area. Once the surface arterioles are dissected, the adjacent anterior bank of the central sulcus is opened, and blood vessels are dissected down to the fundus of the sulcus. This approach disrupts the blood supply to the underlying cortex without mechanically damaging the underlying gray matter [Fig. 1(a)]. After the surgery, monkeys are given two weeks to recover and then re-tested on the HDT for 6 or 12 weeks to assess the rate and extent of impairment of fine motor function.

#### 2.1.3 Brain perfusion and cutting

At the completion of testing, monkeys are euthanized and brains harvested using a two-stage perfusion. First, the deeply anesthetized monkey has a craniotomy over the lesion hemisphere and is then exsanguinated by opening the chest, clamping the descending thoracic aorta, opening the left ventricle, and perfusing ice-cold Krebs’ buffer through the ascending aorta. During this time, the dura is opened and fresh biopsy samples are taken. Then the perfusate is switched to 4 L of warm 4% paraformaldehyde. After perfusion, the brain is blocked *in situ* in the coronal stereotactic plane, removed from the skull, and immersed in cold 4% paraformaldehyde solution for 24 h to ensure complete fixation. The next day, the brain is transferred to a cryoprotectant solution of 0.1 M phosphate buffer with 10% glycerol and then 20% glycerol for several days as described previously.^57^ Once cryoprotected, it is flash frozen in −75°C isopentane and stored at –80°C until removed and cut on a freezing sliding microtome into coronal sections, which are transferred into vials of 15% glycerol and stored at −80°C until thawed and “batch processed.” This process eliminates the risk of freezing artifacts, avoids the shrinkage produced by sucrose cryoprotection^57^ and tissue remains in excellent shape for IHC for at least a decade after final storage.^58^ We have documented that long-term storage after this method does not confound high-quality IHC, ISH, or stereology.^58^ For this study, coronal sections were cut on a freezing sliding microtome and collected for analysis. Sections were collected from the posterior-most aspect of the lesion, through the extent of the lesion, and extended anterior to the lesion to include regions of the CC that are affected by axonal damage projecting from the lesion in the primary motor cortex (M1) [Fig. 1(b)].

### 2.2 Birefringence Microscopy

BRM is a label-free optical imaging technique that enables structural imaging of myelin in post-mortem brain tissue. BRM facilitates two complementary approaches: (1) qualitative imaging using crossed-circular polarizers (CCP-BRM) and (2) quantitative birefringence imaging using a rotating polarizer for illumination, with a circular polarizer as the analyzer before the camera (qBRM). Our implementation of qBRM is described in detail in Blanke and Gray et al.^39^ This system effectively implements 2D polarized-light imaging (2D-PLI) yet, unlike other 2D-PLI setups,^59^^,^^60^ which typically provide only quantitative retardance and optic axis orientation maps, our microscope is optimized to also deliver CCP-BRM for high-contrast qualitative imaging and qBRM for precise, per-pixel retardance quantification in a single, unified platform.

**Fig. 2:**
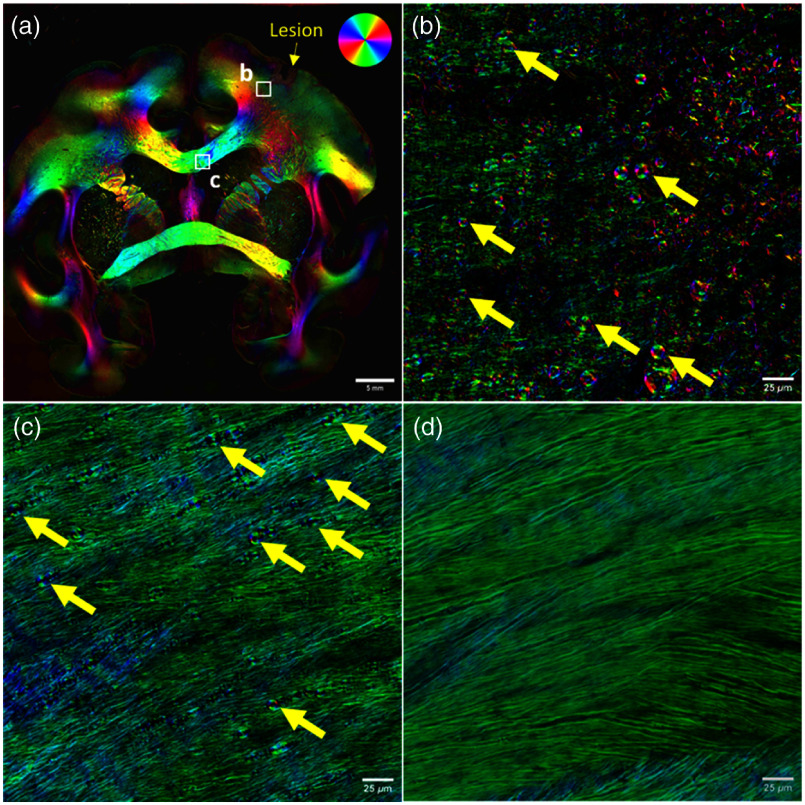
(a) Widefield 4× tile scan of a coronal brain slice from a rhesus monkey with an induced cortical lesion (yellow arrow). qBRM RGB parameter maps are rendered where the brightness of each pixel maps the retardance (myelin density), and the optic-axis orientation (related to fiber direction) is mapped to the color wheel (a—top right). It should be noted that the optic axis of myelin lipids is radial around axons and thus orthogonal to the fiber direction (please refer to Blanke and Gray et al.^51^) and is still meaningful for myelin debris, when there is no longer any axonal structure. (b) High-resolution 40× (NA 0.75) qBRM image of the perilesional gray matter reveals a clear accumulation of myelin debris (yellow arrows). (c) High-resolution 40× (NA 0.75) qBRM image of the corpus callosum, where myelin appears more intact compared to the perilesional region, but still exhibits signs of myelin breakdown (yellow arrows). (d) By contrast, the corpus callosum of a healthy, aging rhesus monkey from the same anatomical region shows little indication of myelin breakdown, demonstrating the specificity of injury-induced myelin pathology visualized by qBRM.

In brief, CCP-BRM enables real-time orientation-independent structural imaging of myelin through a pair of crossed circular polarizers.^48^^,^^61^ This version enables rapid structural imaging of myelin, which can be used to rapidly identify the structural breakdown of myelin. By contrast, qBRM enables the extraction of quantitative parameter maps, including relative retardance (indicative of myelin density) and optic-axis orientation (related to fiber organization) for each pixel in the widefield image. In this version, a series of images is acquired with several (3 or more) angles of illumination of linear polarization and imaged through a circular analyzer. qBRM imaging is modeled using Jones calculus to derive the quantitative parameter maps for relative retardance and optic-axis orientation.^39^^,^^61^^,^^62^ The relative retardance provides an estimate of myelin density, reflecting both the amount of birefringent material and the orientation of myelinated fibers relative to the imaging plane. It is influenced by factors such as fiber inclination, crossing fibers, and local tissue architecture, whereas the optic axis orientation offers complementary information on the spatial organization, or disorganization, of individual myelinated fibers.^39^^,^^51^ qBRM parameter maps are typically visualized as an RGB image where the intensity is mapped by the relative retardance, and the optic-axis orientation is mapped based on a custom colormap [Fig. 2(a) top right].

As previously described in Blanke and Gray et al.,^39^ the custom-built birefringence microscope employs a dual-retarder setup for polarization state generation (PSG) and analysis (PSA). Uniform illumination across the imaging field of view (FOV) is achieved using the Effective Uniform Color-Light Integration Device (EUCLID),^63^^,^^64^ which provides collimated light that passes through polarizing and waveplate components to control the polarization state of illumination. On the detection side, from the objective, light passes through a second set of polarizing and waveplate components to analyze its polarization state before being captured by a high-speed, cooled CMOS camera (Teledyne Iris 9, 9MP @ 16-bit, 30 FPS). The system supports both qualitative imaging (CCP-BRM) and quantitative birefringence imaging (qBRM), with thin-film polarizers and true zero-order quarter-wave plates enabling precise polarization control for both imaging modes. A fast XY and Z scanning stage is used for acquiring tiled image sets across the sample. Images are acquired with a variable overlap and stitched using the FIJI plugin with linear blending.^65^ Images are acquired with either a 40× (NA 0.75; Olympus UPLFLN40XP-2) or a 10X (NA: 0.3; Olympus UPLFLN10XP-2). Images for validation were taken with a 40× objective (lateral resolution: ∼420  nm; axial resolution: ∼2.24  μm), whereas images for widefield analysis of myelin defects were acquired with a 10× objective (lateral resolution: ∼1  μm; axial resolution: ∼14  μm), which provides sufficient resolution for imaging myelin debris in this model (∼1 to 10  μm).

#### 2.2.1 Tissue preparation for birefringence imaging

Brain sections were prepared according to the protocol described by Blanke and Gray et al. (2024).^39^ In brief, previously preserved brain tissue sections are removed from –80°C, thawed to room temperature, and rinsed in 0.1 M tris-buffered solution (TBS) to remove excess cryoprotectant. Sections are floated in a slightly hypotonic buffer (0.05M) and manipulated onto a gelatin-coated microscope slide (50  mm×75  mm) using a fine-tipped paintbrush. The section is then covered by an 85% glycerol solution for refractive index matching to minimize scattering.

#### 2.2.2 Immunohistochemistry and fluorescent labeling

For validation of BRM findings, sections were imaged with BRM before processing for fluorescent labeling and IHC, as several steps for IHC processing can lead to structural damage of myelin.^39^ Tissue is rapidly thawed, transferred onto a microscope slide, and rapidly dried via wicking until adherence is achieved. Slides are coverslipped with 85% glycerol in $H_{2}O$ and kept at 4°C until BRM imaging. After BRM imaging sections were fluorescently labeled on-slide for unaltered 1:1 co-registration of cell nuclei (DAPI), neurofilament (NFL), and fluoromyelin (FM). After BRM imaging, the slides were submerged in buffer, and the coverslip was gently removed. These slides were washed three times in separate baths of buffer solution for 5 min per wash to remove the glycerol solution. The tissue was then washed with 0.5% Triton-X (“Tx”) in Superblock® (Fisher Scientific) for 60 min. The solution was replaced with a fresh bath of 0.5% Tx in Superblock^®^, and the tissue was incubated with primary antibodies for Neurofilament Light (NFL; 1:500), microwaved at 30°C 3 times for 3 min at 300 W, using a PELCO BioWave Pro (Ted Pella, Redding, CA). Sections were incubated in this same solution at room temperature for 8 h, followed by 48 h at 4°C. Sections were then rinsed in 0.1 M buffer for 3 to 6 h, incubated with secondary antibody for 8 h at room temperature, followed by 4°C overnight in solutions for DAPI (1:500) and Fluoromyelin (1:500), and rinsed in 0.1 M buffer for 6 h. Finally, sections were rinsed, mounted, and coverslipped with 85% glycerol solution.

### 2.3 Deep Learning-Based Object Detection

#### 2.3.1 Training annotation and validation for deep learning object detection

Object detection enables precise localization and annotation of objects of various classes within images. In this study, deep learning-based object detection was used to investigate and quantify the spatial distribution and density of myelin defects across different regions of the brain. Deep learning-based object detection learns the spatial features associated with target objects in the training dataset and learns to identify those objects using bounding boxes with pixel coordinates [x, y, width, height]. Object detection was performed using the YOLOv4-Tiny architecture^66^^,^^67^ implemented with the Deep Learning Toolbox in MATLAB R2023b to detect and localize “myelin defect” objects in tiled qBRM images. YOLOv4-Tiny was selected due to its computational efficiency, which can be applied to large-scale images.^66^ To accelerate training and improve performance, the network was initialized with weights pretrained on the Common Objects in Context (COCO) dataset.^68^ These pretrained weights come from a model that has already learned to recognize general patterns and shapes from a large variety of everyday objects (e.g., people, animals, and vehicles), such that the network already identifies features such as edges, textures, and spatial structures. This approach eliminates the need to train the network from scratch and speeds up training of the new network to identify myelin defects in our specialized dataset. The pretrained weights were fine-tuned using a training dataset of manually annotated myelin defects from qBRM images of the corpus callosum in rhesus monkeys with experimentally induced cortical lesions.

Manual labeling of qBRM data was carried out with a custom-built MATLAB annotation software, to handle the large tiled images and different image formats associated with BRM (see Sec. 2.2). Tiled images of the corpus callosum from two monkeys with cortical lesions were manually annotated by two blinded annotators, and only annotations with agreement from both annotators were considered true myelin debris. This method was used to ensure that we maintain high reliability (positive predictive value) after training at the expense of sensitivity (see Sec. 3.2). The manually annotated data across the image were cropped into individual patches of size [128,128,3] for training (Fig. S1 in Supplementary Material 1), leading to a training set size of ∼50,000 images after cropping and augmentation. The training data set was limited to the corpus callosum to demonstrate accurate classification of myelin debris. The CC was chosen as it contains predominantly longitudinal (parallel with the imaging plane), simplifying the necessary analysis pipeline. Further training in other regions is required to accurately identify myelin debris in regions with more complex fiber orientations (see Sec. 3.2).

Data augmentation included standard transformations such as rotation and horizontal/vertical flipping, as well as a novel augmentation approach for qBRM, which we call “color rotation.” In qBRM data, data are stored as RGB images, where the brightness represents relative retardance and the optic-axis orientation is rendered using a custom colormap [Fig. 2(a) top right]. Importantly, the optic-axis orientation is circular in nature, meaning it can be represented cyclically without a defined beginning or end. This allows for cyclic manipulation of the RGB data to generate BRG and GBR images (see Note S1 in Supplementary Material 1), which accounts for variation in tissue orientation due to rotation or flipping of the sample during mounting (Fig. S1 in Supplementary Material 1). Color rotation during the augmentation process ensures the network is robust to changes in sample orientation and is also applied during prediction with test-time augmentation (TTA)^69^^–^^71^ to improve network performance. TTA involves applying data augmentation techniques (color rotation and image transformations) after the model has been trained, during the prediction phase.

Training was conducted in MATLAB with the deep learning toolbox, with pretrained weights from the COCO dataset.^68^ The model was trained for 100 epochs using the Adam optimizer with an initial learning rate of 0.005 and reduced by a factor of 2 every 10 epochs following a step decay schedule and a mini-batch size of 256. A custom testing dataset from three different monkeys and 5 to 15 different tissue sections for each monkey was used to assess network performance compared with manual annotation. For inferencing, the trained model is applied using the Slicing Aided Hyper Inference (SAHI) techniques^72^^–^^74^ to enable detection of small objects in large images. SAHI works by dividing the input image into smaller overlapping tiles, in our case, 128×128  pixels with 50% overlap, allowing the object detection network to identify smaller objects within a larger FOV image. Bounding box predictions are accumulated with SAHI and TTA, and non-max suppression (NMS) is applied to eliminate redundant predictions by retaining only the highest-confidence predictions for overlapping boxes. In the future, more sophisticated voting techniques, as opposed to simple NMS, can be used to enhance the accuracy for object detection.^75^

#### 2.3.2 Automated detection and quantification of myelin debris in the corpus callosum using YOLOv4-tiny

**Fig. 4:**
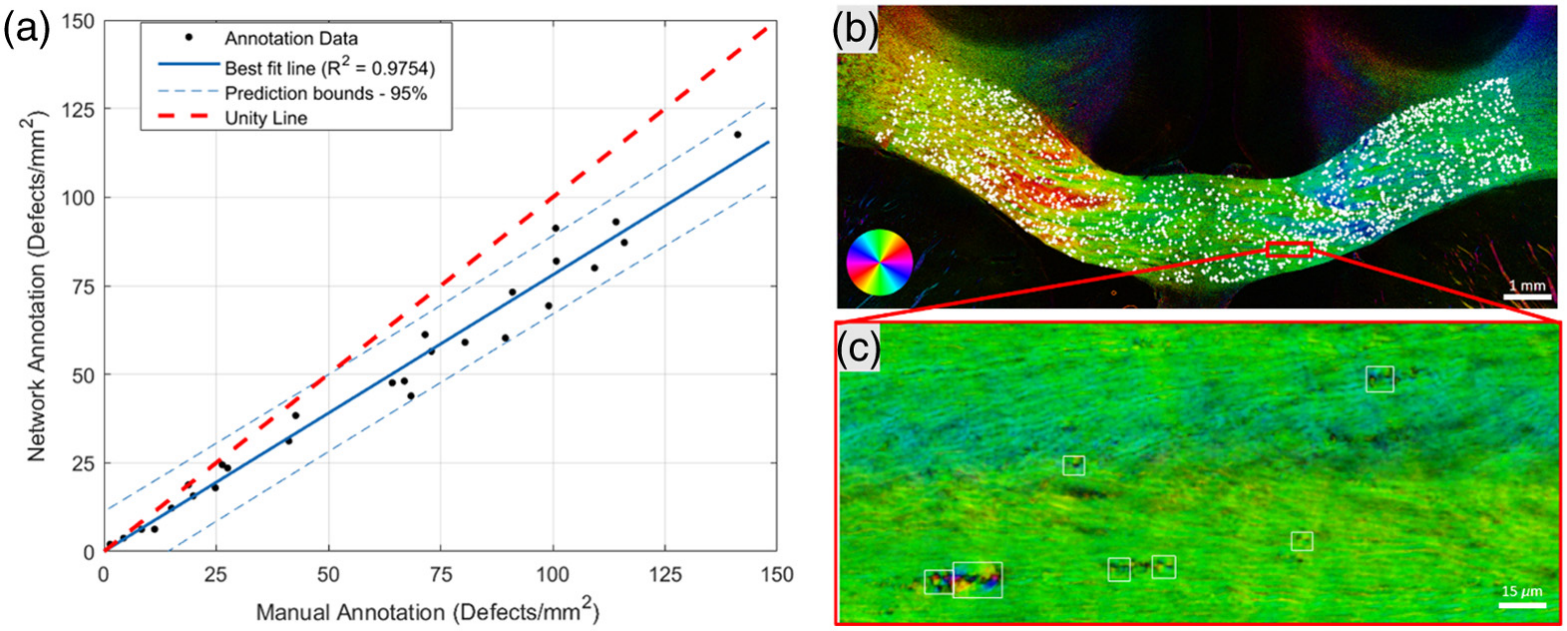
Validation of automated identification and spatial distribution of myelin debris with deep learning-based object detection with YOLOv4 Tiny network. (a) A test data set taken from 3 monkeys with 5–15 sections from each monkey was taken and blindly annotated to assess the performance of the network compared with manual annotation. A high linear correlation between the manual annotation and network annotations ($R^{2}=0.9754$) highlights that data annotated by the trained network is representative of the myelin damage within the corpus callosum. (b) A representative example of a 10× tiled image of the corpus callosum with network-detected myelin debris (white boxes) shows the network’s ability to identify myelin debris in this model. (c) A zoomed-in panel of the 10× tiled image set showing examples of myelin debris identified by the trained object detection network.

To assess myelin damage resulting from the cortical lesion, we quantified myelin debris density within the corpus callosum through the extent of the lesion and between monkeys with different post-injury recovery times. The analysis of individual corpus callosum sections was conducted using the workflow outlined in Fig. S4 in Supplementary Material 1. First, high-resolution images of the corpus callosum were acquired at 10X (NA: 0.3) magnification and stitched. The trained object detection network then annotated myelin defects within the stitched images using TTA and a sliding window, as described in Sec. 2.3.1. To quantify the degree of myelin damage, a manually drawn region of interest (ROI) of the corpus callosum was used to normalize defect counts to the total analyzed area, to establish a defect density. In a single brain section, the distribution of myelin debris within the corpus callosum appeared relatively homogeneous [Fig. 4(b)]; therefore, we represent each section with a single value corresponding to its myelin defect density within the corpus callosum. Automated analysis was performed using qBRM images, as the object detection network was pretrained on RGB inputs; however, the same pipeline can be readily adapted for grayscale CCP-BRM data to enable faster acquisition and broader applicability.

## 3 Results

### 3.1 BRM Identifies Myelin Degradation in Rhesus Monkey Model of Circumscribed Cortical Injury

In this study, we investigate structural changes to myelinated fibers in a rhesus monkey model of cortical injury, in which a lesion was induced in the hand representation area of the primary motor cortex (M1) [Fig. 2(a)]. This injury results in local, large-scale cell death and the accumulation of both cellular and myelin debris in the area adjacent to the lesion, with axonal and myelin degeneration extending into the surrounding perilesional cortex.^76^^,^^77^ Our previous study using qBRM demonstrated the presence of myelin debris in the perilesional gray matter;^48^ but did not assess the distribution and extent of myelin pathology beyond the immediate injury site or identify the structural makeup of these myelin defects. Here, we build upon this work by leveraging the high-throughput capabilities of qBRM to systematically map myelin damage across entire brain sections, allowing us to capture both localized and distal effects of cortical injury.

Consistent with prior findings, qBRM imaging confirms widespread myelin debris accumulation in the perilesional cortex [Fig. 2(b)]^48^^,^^53^^,^^76^ However, beyond this localized damage, we also observed myelin breakdown in the corpus callosum [Fig. 2(c)], a major white matter tract, which facilitates interhemispheric communication.^78^^,^^79^ This downstream degradation, consistent with Wallerian degeneration, occurs as axons disconnected from their cell body by the lesion site undergo progressive deterioration, reducing trophic support from the injured axons and ultimately leading to myelin degeneration.^9^ Unlike the highly fragmented and dense myelin debris seen in the perilesional space, the damage in the corpus callosum appears more dispersed and subtle, characterized by vesiculated birefringent structures indicative of progressive myelin breakdown [Fig. 2(c)]. By contrast, the corpus callosum of a normal aging monkey without cortical injury [Fig. 2(d)] shows no comparable signs of myelin disruption, suggesting that most of the observed pathology in the injury model is lesion-induced rather than age-related damage.^13^^,^^17^^,^^30^

A major advantage of BRM over conventional imaging methods is its ability to rapidly image large areas at high resolution, making it uniquely suited for studying widespread myelin pathology. Our BRM microscope enables imaging of an entire rhesus coronal brain section (∼15 to $25\;{\mathrm{cm}}^{2}$) with qBRM at ∼1  μm resolution in under 2 h, with targeted imaging of the corpus callosum (∼60 to $100\;{\mathrm{mm}}^{2}$) completed in under 5 min. Achieving a similar throughput with traditional fluorescence-based imaging techniques, or other laser scanning techniques, would be significantly more time-consuming and less practical, as these methods require staining, adding considerable time and complexity to the workflow. By enabling a global assessment of myelin integrity across entire brain sections, BRM not only confirms perilesional myelin damage but also reveals more subtle, long-range degenerative changes that would be challenging to identify with conventional imaging approaches.

**Fig. 3:**
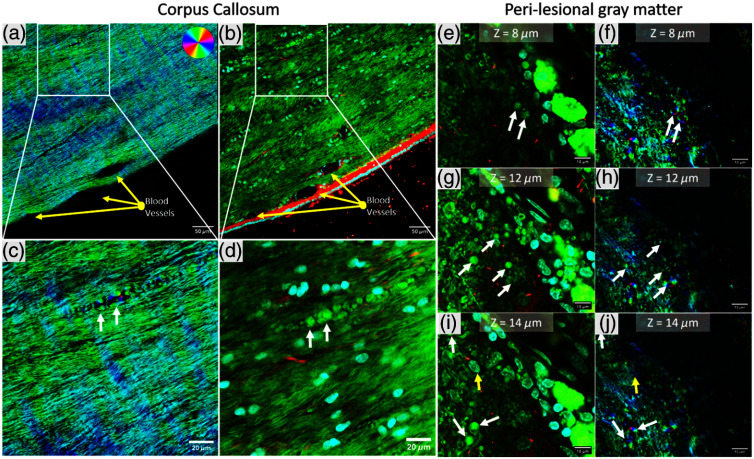
Validation of BRM-detected myelin debris using immunohistochemistry (IHC) and FluoroMyelin staining in a rhesus monkey cortical injury model. (left panels, a–d) Images from the corpus callosum show BRM-identified myelin debris (a, c), highlighted in a zoomed-in region (white box) as vesiculated myelin (white arrows). These structures are confirmed with fluorescent staining (b, d): DAPI (blue) confirms that the structures do not colocalize with cells, neurofilament (green) indicates that they are not consistent with axonal damage, and FluoroMyelin (white arrows) confirms they are vesiculated myelin debris. (right panels, e–j) Images from the perilesional gray matter demonstrate the same phenomenon with qBRM (f, h, j) and fluorescence imaging (e, g, i). The z-stack images across different focal planes (Z=8.12 and 14  μm) through the 30-μm tissue section. These z-stack images confirm the presence of smaller myelin debris detected by both BRM and fluorescent staining (white arrows) and that BRM is not sensitive to cellular structure (yellow arrow). Raw z-stack files for the IHC validation are available in Supplementary Material 2 and Supplementary Material 3.

To validate the structural makeup of the myelin debris detected by qBRM, IHC labeling and FluoroMyelin staining were performed on cortical injury whole-brain sections. These were first imaged with qBRM in both the perilesional gray matter and the corpus callosum, followed by on-slide staining of the same undisturbed tissue section with DAPI (cell nuclei), Neurofilament light (NFL; axonal marker), and FluoroMyelin (lipophilic myelin dye; ThermoFisher #F34651). Co-registration of the qBRM and fluorescence images revealed that structures identified by qBRM as myelin debris correspond to vesiculated myelin, confirmed by FluoroMyelin staining (Fig. 3, white arrows). It should be noted that due to the widefield nature of BRM, which lacks optical sectioning, it may miss small or faint myelin debris that are more readily detected using confocal-based FluoroMyelin imaging. This was observed in both the corpus callosum [Figs. 3(a)–3(d)] and the perilesional gray matter [Figs. 3(e)–3(j)]. In addition, DAPI staining showed no overlap between these myelin debris structures and cell nuclei, confirming that qBRM is not sensitive to cellular components [Figs. 3(i) and 3(j), yellow arrow]. Furthermore, NFL labeling demonstrated that these myelin accumulations do not contain axonal debris, indicating that they primarily consist of fragmented myelin rather than degenerating axons. In the corpus callosum, which predominantly consists of longitudinally aligned axons, transverse fibers are rare, so spherical birefringent structures with radially varying optic axes are unlikely to represent intact myelin. In the perilesional cortex, high-resolution z-stack imaging showed that these structures appear in only one to three optical planes, and FluoroMyelin co-staining confirms their identity as isolated vesicles, as opposed to transverse axons, which would traverse the entire thickness of the tissue. Critically, NFL staining revealed no axonal signal within these structures, confirming they are not transverse axons but indeed represent extracellular myelin debris. However, it should be noted that this myelin debris is likely a direct consequence of axonal degeneration in fibers projecting from the lesion site through this region of the corpus callosum. For further comparison, we utilized SCoRe microscopy to image the presence of myelin debris in this model as shown in Fig. S2 in Supplementary Material 1. In the perilesional gray matter, SCoRe imaging revealed a near-total absence of intact myelinated fibers. However, its ability to identify myelin debris was limited due to the loss of reflectance signal when the axonal multilayer myelin interface is disrupted (the main contrast mechanism of SCoRe) (Fig. S2 in Supplementary Material 1). Similarly, although intact myelinated fibers were readily observed in the corpus callosum using SCoRe, the detection of myelin debris was limited due to the absence of a strong reflectance signal in fragmented myelin structures (Fig. S3 in Supplementary Material 1). This highlights that the loss of the SCoRe signal indicates structurally damaged myelin but does not directly image the altered myelin itself. Because a lack of signal could also result from the complete absence of a myelinated axon at that location, this introduces some ambiguity in interpreting SCoRe imaging and quantification of true structural alterations.

### 3.2 BRM Enables Automated Analysis of the Spatial Distribution of Myelin Defects Across Large Brain Sections

We have demonstrated that BRM is highly sensitive to detecting myelin debris in a rhesus monkey model of cortical injury (see Sec. 3.1). To quantify the spatial distribution of myelin defects (pathology) across brain sections, we trained a YOLOv4-Tiny network to automate myelin debris identification in high-resolution (∼1  μm resolution) BRM images. For network training, we focused exclusively on images from the corpus callosum (CC). Although BRM analysis can be expanded to other regions of the brain (assuming adequate training in these regions), the CC was chosen for several reasons: (1) It is of significant interest in neurodegeneration research due to its vulnerability in various models of damage and disease.^12^^,^^80^^,^^81^ (2) Its fiber architecture is relatively homogeneous, with fibers predominantly aligned in the same direction, simplifying the necessary analysis pipeline. A training set from three sections (three separate monkeys) was manually annotated by two blinded annotators and used for network training (see Sec. 2.3.1). The network’s prediction performance, after training, was evaluated on a subset of brain sections, ranging from 5 to 15 from three different monkeys depending on tissue availability, each exhibiting varying degrees of myelin damage. A uniform sampling of the corpus callosum from each of these sections was carried out to cover ∼5% to 10% of the total CC area, and blinded manual annotation was compared with network annotations on these images [Fig. 4(a)]. Each point in Fig. 4(a) represents a single uniformly sampled subset of images from the full widefield image of the corpus callosum (∼12  mm×∼5  mm). The y-axis shows the average defect density across the CC predicted by the network, and the x-axis shows the corresponding manual annotation for that region. Because multiple sections were sampled per animal, the figure reflects variation both within and across biological replicates. The trained model achieved a sensitivity (true positive rate) of 77.4% and a positive predictive value (precision) of 88.9%, with a very low false positive rate (8.7%). Although the network does not capture every instance of myelin debris present in the images, its low false positive rate ensures that detected defects represent true pathology rather than artifacts. Importantly, because the true positive rate is high and the false positive rate is low, the overall defect density identified by the network is linearly correlated with manual annotations ($R^{2}=0.9754$) [Fig. 4(a)]. This suggests that the network provides a consistent and representative quantification of myelin pathology across different sections and monkeys. It should be noted that the accuracy of the network degrades outside of the CC, as there are fibers of complex orientations that lead to a higher false positive rate. To enhance the network application across other white- or gray-matter regions, further training and validation are required.

**Fig. 5:**
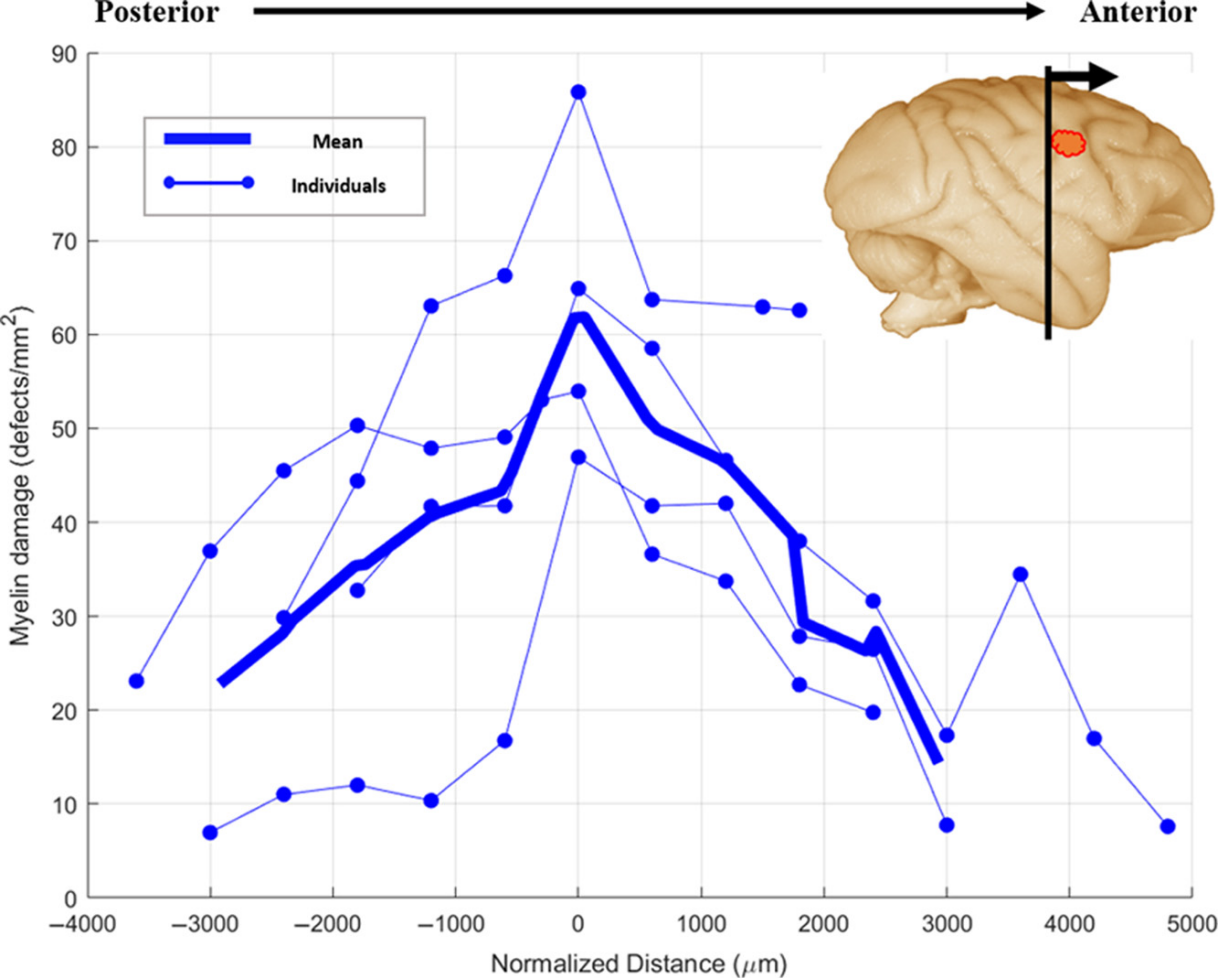
Quantification and spatial distribution of myelin damage across the corpus callosum and through the extent of the lesion. Quantification of myelin damage across coronal sections taken at 600  μm intervals through the extent of the lesion in monkeys 12 weeks post-injury. The plot shows individual data points for the average density of myelin debris in the corpus callosum for each monkey (thin lines) and the group mean (thick line). Distance is normalized to the maximum damage across the lesion to account for individual differences in anatomy, lesion location, and slice position. A clear increase in myelin damage is observed at the center of the lesion across all monkeys, corresponding to the region where the most axonal projections from the cortical lesion traverse the corpus callosum.

By leveraging this automated pipeline, we mapped the spatial distribution and density of myelin debris across multiple brain sections, allowing us to examine patterns of damage within the corpus callosum [Figs. 4(b) and 4(c)] and through the rostro-caudal extent of the lesion (Fig. 5). We quantified defect density as the number of debris elements per unit area rather than total defect area, reflecting the bounding-box outputs of our deep-learning model and the challenges of achieving pixel-level accuracy, especially for irregularly shaped defects. The analysis of individual corpus callosum sections was conducted for sections taken along the rostro-caudal extent of the lesion and was represented as a single value corresponding to the myelin defect density across the CC (see Sec. 2.3.2). Notably, we observed occasional false-positive detections in regions adjacent to the corpus callosum, caused by circular birefringent features (e.g., transverse and oblique axons) that mimic debris (Figs. S4 and S6 in Supplementary Material 1). To eliminate these artifacts, all quantitative analyses were confined to a manually drawn ROI around the CC. We acknowledge that this manual masking step reflects a current limitation of our network; future work will focus on training a more generalized model capable of whole-brain myelin defect detection without ROI preselection (see the Discussion section). Examples of true positive, false positive, and false negative annotations of myelin debris by the network are provided in Fig. S5 in Supplementary Material 1. Analyzing serial sections spanning the lesion at 600-μm intervals (Fig. 5), we observed a distinct increase in defect density when passing through planes of the CC aligning with the location of the lesion associated with the most axonal projections from M1. This pattern reflects a higher density of axonal projections from the cortical injury site (M1) through this region of the corpus callosum.^82^ To account for anatomical variability between monkeys, damage location was normalized relative to the position of maximum myelin defect density. Importantly, a normalized distance of 0  μm does not necessarily correspond to the physical center of the lesion, but instead represents the region where axonal projections from the cortical injury were most dense within the corpus callosum. In all subjects, visual inspection confirmed that the maximum damage occurred within a consistent anterior/posterior anatomical position within the corpus callosum, supporting the assumption that this region represents the primary site of lesion-related callosal projections.

This approach enables a three-dimensional visualization of the distribution of myelin damage, which would be challenging to achieve using imaging modalities with lower throughput (EM, SCoRe, IHC) or with insufficient resolution such as diffusion MRI.^83^ In addition, although the current neural network was trained exclusively on images from the corpus callosum, we applied it beyond the corpus callosum to detect myelin damage in other areas, including the perilesional cortex and surrounding white matter on both the lesional and contralateral hemispheres (Fig. S6 in Supplementary Material 1). Blanke et al.^48^ demonstrated that there was minimal damage in the contralateral hemisphere, which acts as a control to quantify relative changes between the injured hemisphere and the contralateral hemisphere. It was found that the trained network detects additional structures such as oblique or transversely oriented myelin sheaths, which have similar features to myelin pathology and contribute to false positives (see the Discussion section). In addition to the false positives from transverse axons, some features shown in Fig. S6 in Supplementary Material 1 are imaging-related artifacts. The characteristic blockiness in the upper-left tile arises from imperfect objective focusing over the large region of tissue for stitching (with minimal overlap), whereas the stripe-like artifacts in the lower-right quadrant are due to localized tissue wrinkling. Future incorporation of automated focus correction or dynamic focus-stacking during high-resolution tile acquisition could substantially mitigate these artifacts and improve specificity. Nonetheless, despite these misclassifications, statistically, the network provides a relative measure of damage density, revealing a higher density of detected defects in the lesional hemisphere compared to the contralateral side (Fig. S6 in Supplementary Material 1). Future refinements, including higher-resolution training data and volumetric imaging, could further optimize the network for whole-brain analysis while reducing false positives in other regions of the brain.

### 3.3 Quantification of Myelin Debris as a Function of Post-injury Recovery Time

**Fig. 6:**
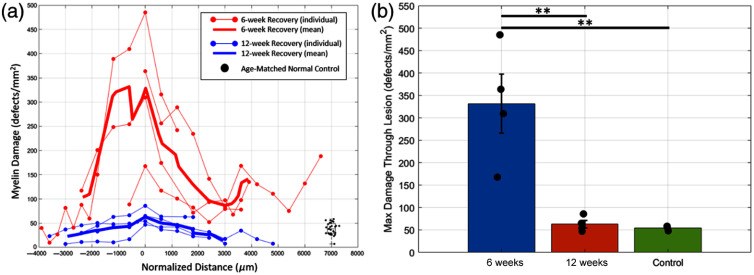
Quantification of myelin damage across the lesion and summary of maximum damage per monkey (a) Individual myelin damage profiles across the lesion extent for each monkey (thin lines with dots) in the 6-week recovery group (red) and the 12-week recovery group (blue—data replicated from Fig. 5). The mean myelin damage for each group (N=4) is shown as a thick red or blue line, respectively. Age-matched normal controls (N=4) are represented as a box plot on the right (black). Myelin damage is quantified as the density of myelin debris detected by the object detection network across the corpus callosum for each section, with normalized distance plotted from posterior to anterior (negative to positive). (b) Maximum myelin damage through the lesion for each monkey, showing a significant difference between the 6-week and 12-week recovery groups (**P<0.01), as well as between the 6-week recovery group and age-matched controls (**P<0.01). No significant difference is observed between the 12-week recovery group and the age-matched controls, suggesting that myelin debris clearance is largely complete by 12 weeks, aligning with functional recovery timelines. Statistical significance was determined using a one-way ANOVA followed by Tukey’s Honestly Significant Difference (HSD) test for multiple comparisons. Bars represent group means ± standard error of mean, with individual data points shown.

We have demonstrated that qBRM, combined with deep learning-based object detection, effectively visualizes myelin damage within the CC resulting from an induced cortical lesion. To quantify myelin repair and clearance as a function of post-injury recovery time, we analyzed tissue from monkeys that recovered for either 6 or 12 weeks following cortical injury prior to tissue harvest. Although previous studies have shown that functional recovery plateaus around 6 to 8 weeks post-injury, the extent of underlying myelin repair had not been quantified. We quantify the myelin damage in the CC of 8 to 15 sections per monkey (depending on tissue availability), spaced in increments of 300 to 600  μm apart, for regions of the corpus callosum associated with the cortical injury (Fig. 5). We compared four monkeys (N=4) per recovery group (6 or 12 weeks) to age-matched controls (N=3) with no injury. Our findings reveal that myelin damage in the 6-week recovery group was significantly higher than in both the 12-week recovery group (p<0.01) as well as compared with the age-matched controls (p<0.01) [Fig. 6(b)]. Because there was no significant difference between the 12-week recovery group and the controls, this suggests that myelin pathology is largely cleared by 12 weeks, consistent with functional recovery timelines observed in prior studies.^53^ Interestingly, the 6-week group exhibited higher mean myelin damage but greater variability between individual monkeys, likely reflecting differences in the ongoing healing and recovery process. This variability may stem from individual differences in the extent of initial lesion damage, anatomical variation, and variability in the biological mechanisms underlying myelin repair.

## 4 Discussion and Conclusion

### 4.1 Summary

Here, we utilized BRM to study structurally altered myelin in a rhesus monkey model, where myelin degeneration was produced by a circumscribed cortical injury. Using our customized birefringence microscope, we acquired whole hemisphere montages of 30-μm-thick sections imaged at 10× magnification (NA: 0.3) with an IRIS-9 camera. We also show that BRM enables high-throughput structural imaging and detection of myelin and myelin defects. We demonstrate the ability of BRM to identify intact as well as damaged myelin across large brain areas and validate their structural makeup with IHC (Neurofilament) and fluorescent staining (FluoroMyelin^®^ and DAPI). BRM enables rapid characterization of structurally altered myelin across large brain sections. We then trained and validated a deep learning object detection model for the automated identification of myelin pathology in this model to study the spatial distribution of myelin damage across the entire corpus callosum and across sections that pass through the extent of the corpus callosum, where the damaged fibers cross. We then investigated the differences in myelin damage present in the CC for monkeys with varying recovery times. We observed an abundance of myelin damage at 6 weeks post-injury, but markedly reduced levels of damage in the corpus callosum after 12 weeks of recovery. These results demonstrate that BRM can enable large-scale investigations into the spatial distribution and extent of myelin damage within the corpus callosum. With appropriate characterization and training, this technique can be adapted to other brain regions and disease models, enabling high-throughput, quantitative analysis of myelin structure across conditions, increasing sample sizes, and supporting broader investigations.

### 4.2 Relative Merits of BRM Compared With Other Myelin Imaging Methods

As discussed, BRM is a widefield (camera-based), label-free imaging technique that enables high-throughput ($∼\text{several}\;{\mathrm{mm}}^{2}$), high-resolution (∼1  μm) imaging of myelin structure, resolving down to individual myelinated fibers. Unlike other imaging modalities, BRM and other PLI techniques can efficiently provide quantitative information over large areas of tissue (and several tissue sections) that is otherwise unfeasible with other high-resolution techniques. Importantly, BRM does not require staining, reducing sample preparation time and avoiding variability introduced by labeling efficiency. By contrast, FluoroMyelin and other labeling methods require both arduous staining procedures and confocal image acquisition, making it significantly slower and less scalable for large-area quantification, even with the potential increase in sensitivity with these labels. One of the most widely used techniques for large-scale *in vivo* brain imaging is diffusion-weighted MRI (dMRI), which is a microstructural imaging technique sensitive to the anisotropic diffusion of water confined by microstructural barriers such as myelin sheaths, axonal membranes, and other cellular structures. dMRI-derived metrics (e.g., fractional anisotropy and radial diffusivity) have been correlated with pathology in stroke,^81^^,^^84^ aging,^85^ and neurodegeneration, including AD,^86^ CTE,^23^ and multiple sclerosis (MS).^87^ dMRI is invaluable for *in vivo* longitudinal studies, allowing researchers to track structural changes over time.^88^ For a more direct myelin-specific contrast, several other MRI approaches have been developed: myelin water fraction (MWF), which isolates short T2 signals from water trapped between myelin bilayers; magnetization transfer imaging (MTI); and quantitative susceptibility mapping (QSM), which maps tissue magnetic susceptibility.^89^ However, the limited spatial resolution of these techniques makes them less suited for detecting or revealing microscopic myelin alterations, particularly in regions with complex fiber orientations. Modern dMRI pipelines can estimate multiple fiber orientations, but accuracy is constrained by voxel volume and modelling assumptions.^90^ Albeit limited to *ex vivo* imaging, BRM offers a high-resolution alternative that complements dMRI by providing detailed structural information. For example, BRM can visualize vesiculated myelin debris not resolved by dMRI, providing insight into ultrastructural changes that may underlie signal alterations in affected brain regions. Previous research for validation of myelin imaging across spatial scales has utilized lower resolution myelin imaging techniques, including polarization-sensitive optical coherence tomography (PS-OCT)^51^ and PLI,^91^^,^^92^ to assess myelin content in both white and gray matter across microscopic and mesoscopic scales. Given its higher resolution, BRM is also complementary to PS-OCT.

Furthermore, BRM serves as a non-destructive, label-free imaging tool that can be used alongside other high-resolution myelin imaging modalities. These include both label-free techniques (e.g., SCoRE microscopy, two-photon microscopy (2 PM), and third-harmonic generation (THG) imaging) and fluorescent label-based methods (e.g., FluoroMyelin, immunostaining for MBP, dMBP, and MAG). We have previously demonstrated that common tissue processing techniques can alter underlying myelin structure, potentially introducing artifacts in imaging.^39^ By contrast, BRM preserves the native lipid architecture of myelin, allowing for a more accurate representation of myelin integrity. At the highest resolution, EM remains the gold standard for ultrastructural characterization of myelin. However, EM can only examine small regions of tissue. Moreover, EM is labor-intensive, often expensive, requires hazardous chemicals, and demands highly specialized expertise, limiting its applicability. Athough BRM cannot achieve EM’s nanoscale resolution due to the diffraction limit of visible light, it provides a scalable and accessible method for assessing myelin degradation over large fields of view.

Overall, BRM enables large-scale, high-throughput assessment of myelin integrity at the individual fiber level, providing a crucial link between high-resolution optical imaging techniques (e.g., SCoRe, THG, 2PM, and label-free microscopy) and mesoscopic or macroscopic imaging modalities (e.g., PS-OCT, dMRI, and PLI). Although BRM does not provide imaging at the nanoscopic resolution of EM, it can enable larger-scale assessments of structural alterations at spatial scales that are well-suited for optical imaging. Thus, BRM has the potential to offer new insights into the ability to study myelin pathology for a variety of disease models and spatial scales, which can lead to further quantitative insights that complement existing imaging modalities.

### 4.3 Expanding the Applications of BRM in Neuroscience (Neurodegeneration, Aging, etc.)

Myelin degradation or dysfunctional structural alteration to myelin is frequently found in conjunction with many common neurological diseases, such as AD),^18^^,^^21^ CTE,^23^ and multiple sclerosis (MS).^15^^,^^16^^,^^87^ Myelin degradation is also found to accumulate during normal aging.^17^^,^^32^^,^^34^^,^^93^ Although the etiology of myelin damage in these contexts remains incompletely understood, emerging evidence suggests that myelin damage may be a critical but understudied factor in disease progression. For instance, the longstanding hypothesis that amyloid beta deposition is the main instigator of AD progression has faced increasing scrutiny,^94^^,^^95^ prompting a shift toward exploring alternative pathways, such as vascular dysfunction and its impact on myelin integrity.^96^^,^^97^ Although BRM can only be applied on a postmortem basis, its ability to visualize myelin defects in detail can be applied to assess the extent and distribution of structural degradation, as well as to investigate the effects of therapeutic interventions for the clearance of myelin debris or repair of structurally altered myelin.^53^^,^^76^^,^^98^ Thus, we plan to extend our BRM studies to evaluate the efficacy of a novel therapeutic, mesenchymal stem cell-derived extracellular vesicles (MSC-EVs).^53^^,^^76^^,^^77^ We have shown that MSC-EVs facilitate recovery of motor function after cortical injury, raising the possibility that it may enhance myelin repair (or promote clearance of damaged myelin)^76^^,^^77^ as well as reducing inflammatory damage.^99^^–^^102^ BRM will enable us to study the effects of myelin damage and subsequent repair across entire brain sections

### 4.4 Limitations and Challenges

Although BRM provides rapid, label-free imaging of brain tissue, there are several considerations for analyzing myelin changes across different species and brain regions. BRM suffers from similar constraints as widefield fluorescence, where out-of-focus signal and optical scattering obscure imaging. Due to this limitation, tissue must be no greater than 100-μm thick for imaging in gray matter and is optimally no more than ≤30  μm for imaging in dense white matter tracts to ensure sufficient contrast for all myelinated structures. Thicker sections result in reduced clarity due to light scattering and the accumulation of birefringent signals from multiple focal planes. These limitations may be alleviated by incorporating structured illumination techniques, which improve optical sectioning by rejecting out-of-focus light,^103^^,^^104^ thus enabling imaging of thicker tissue sections and improving volumetric imaging with BRM. In addition, one of the major limitations of BRM is the need to characterize myelin damage across diverse brain regions. These differences in fiber architecture influence the appearance of structurally intact myelin, as compared with healthy myelin with diverse orientation, and may require retraining of the deep learning detection models for reliable identification and quantification, potentially by using volumetric imaging at higher resolution to differentiate between healthy myelin and debris structures. Ongoing efforts are focused on addressing these challenges to extend the applicability of BRM across a broader range of tissue types, brain regions, and disease models, ultimately improving its utility for studying structural myelin pathology at scale.

### 4.5 Conclusion and Future Directions

BRM enables label-free detection of structural changes to myelin, providing a powerful tool for quantifying myelin pathology across multiple brain sections. Here, we demonstrate that BRM effectively identifies myelin pathology in a rhesus monkey model of cortical injury, but this work can be extended to study myelin degradation in a wide variety of models for neurodegeneration, trauma, and aging. Our approach offers a scalable method for characterizing myelin pathology and for assessing the efficacy of different therapeutic treatments. Future studies will look to extend the reach of BRM to study myelin damage in various contexts and investigate novel methods for myelin repair and recovery. This will require characterization of myelin changes across different models of disease, injury, and aging, similar to what was carried out in the present study. As with any imaging technique, accurate assessment requires thorough characterization and validation of the structures of interest. Expanding BRM studies to additional brain regions and investigations of myelin degradation in other disease models will require further characterization and validation to distinguish different types of myelin alterations associated with various conditions, such as autoimmune damage in multiple sclerosis. With proper characterization and training, BRM, paired with a generalized deep learning network, has the potential to facilitate studies of myelin degradation across large tissue sections from a range of disease, injury, and aging models in various species ranging from humans and monkeys, down to rodents.

## Supplementary Material

10.1117/1.NPh.12.4.045006.s01

10.1117/1.NPh.12.4.045006.s02

10.1117/1.NPh.12.4.045006.s03

## Data Availability

All code used for the acquisition and analysis of qBRM data can be found at https://github.com/alexgray103/BiRScope. All other imaging data are available upon request, as the underlying data are too large for most data repositories (>500  GB).
