# Supplementary material for: Birefringence microscopy enables rapid, label-free quantification of myelin debris following induced cortical injury
Source: Neurophotonics. 2025 Oct 28;12(4):045006. doi: 10.1117/1.NPh.12.4.045006 (PMC12576696; doi:10.1117/1.NPh.12.4.045006)
Supplement: Supplementary file 1 [file NPh_012_045006_SD001.pdf]

Birefringence microscopy enables rapid, label-free quantification of myelin debris following induced cortical injury

Alexander J. Gray [a], Rhiannon E. Robinson [b], Samer Berghol[a], Douglas L. Rosene[b,d], Tara L. Moore[b,d], Irving J. Bigio[a,c,*]
[a] Department of Biomedical Engineering, Boston University College of Engineering, Boston, MA, USA
[b] Department of Anatomy and Neurobiology, Boston University School of Medicine, Boston, MA, USA
[c] Department of Electrical and Computer Engineering, Boston University College of Engineering, Boston, MA, USA
[d] Center for Systems Neuroscience, Boston University, Boston, MA, USA
***Corresponding Author, E-mail: algray@bu.edu**

Supplemental note S1: **RGB representation of qBRM data and color augmentation**

Quantitative birefringence microscopy (qBRM) enables the extraction of structural parameter maps that can be used to assess myelin integrity and detect pathological changes. qBRM data can be visualized in an RGB format, where brightness represents relative retardance (a proxy for myelin density), and color corresponds to optic-axis orientation (related to fiber direction) using a custom color wheel. This representation provides a comprehensive view of myelinated axons, illustrating both their density and structural orientation in a single image. The optic-axis orientation data in qBRM is inherently circular, meaning there is no fixed starting point for its representation. While the starting value can be adjusted prior to RGB conversion, it can also be modified post-processing by rotating the RGB channels. Specifically, shifting the RGB channels to BRG or GBR maintains the same relative retardance (myelin density) while altering the color mapping of fiber orientation.

In the context of deep learning, this color augmentation serves a crucial role in reducing training bias (overfitting the training data) and improving generalization for prediction of new samples with potentially different sample orientation. Tissue sections may be mounted at varying angles or flipped during sample preparation, which introduces variability in the network's training. By rotating RGB channels, we effectively simulate different orientations of the optic-axis data without altering the underlying structure of the sample. This augmentation strategy helps the object detection network learn robust representations of myelin, ensuring consistent performance across diverse brain regions and sample preparations.

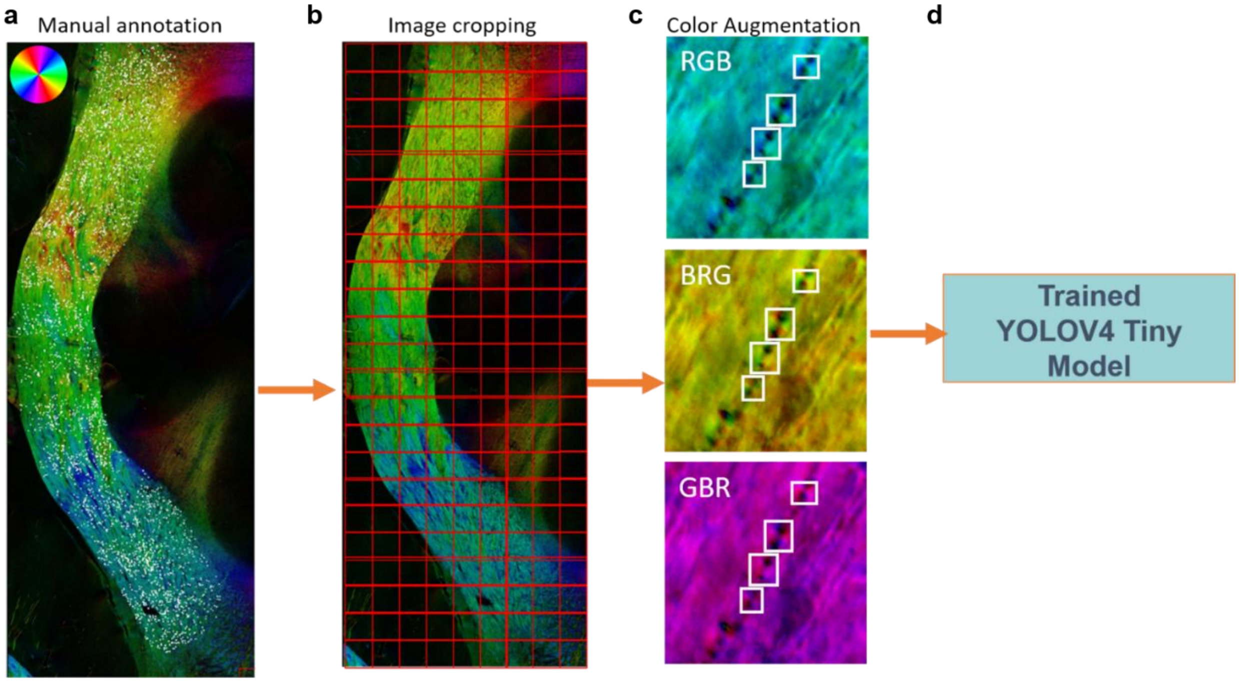

Figure S1: YOLO-v4 tiny training pipeline for automated identification of myelin debris. (a) Custom built annotation software in MATLAB is used to annotate training images of the corpus callosum from cortical injury monkeys (left- white boxes). (b) Images are then cropped (with 50% overlap) to the intended network size of [128,128,3]. (c) Color augmentation is used to account for variation in sample orientation. The underlying orientation data is circular in nature and thus rotated to account for changes in sample orientation. To accomplish this, processed RGB images are augmented to other colorways (orientation) such as BRG and GBR to simulate changes in sample orientation and varying directions across the corpus callosum to improve network generalization (See supplementary note 1). (d) These images along with other traditional augmentation techniques are used to train a YOLO-v4 tiny network.

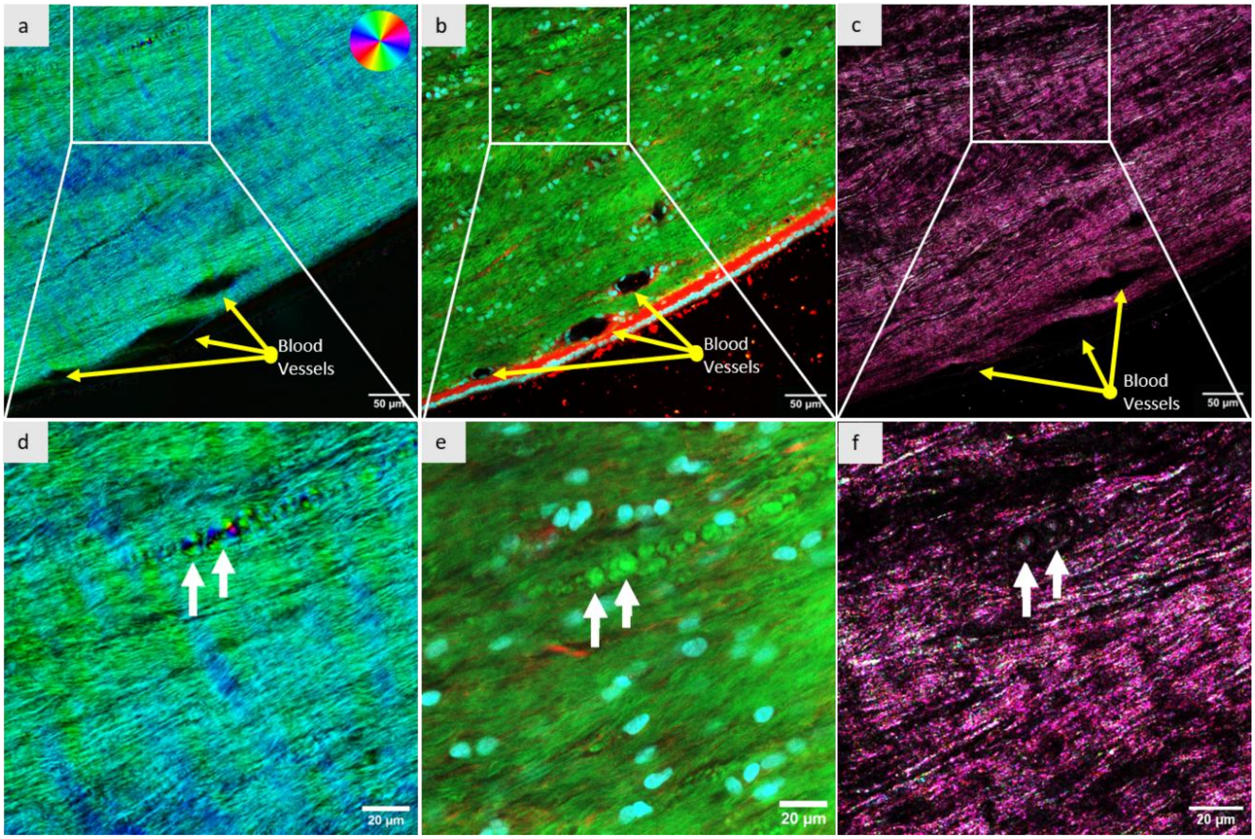

Figure S2: Comparison of vesiculated myelin as imaged by BRM, fluorescent labeling (DAPI – Cyan;, Fluoromyelin (green) and Neurofilament (red)) and SCoRe imaging within the corpus callosum of a rhesus monkey with an induced cortical injury (Fig. 1a). Widefield views and zoomed-in regions are shown for BRM (a, d), fluorescence imaging with IHC (b, e), and SCoRe (c, f). In the fluorescent panels (b, e), myelin is labeled with FluoroMyelin (green), axons with Neurofilament (red), and nuclei with DAPI (cyan). White arrows indicate regions of vesiculated myelin, which are identified with both BRM (d) and IHC (e) due to their disrupted structure. These same features are not easily visualized in SCoRe images (f), likely due to the loss of reflectance in vesiculated myelin structures. Yellow arrows indicate prominent blood vessels that serve as anatomical landmarks across modalities for alignment of the imaged fields.

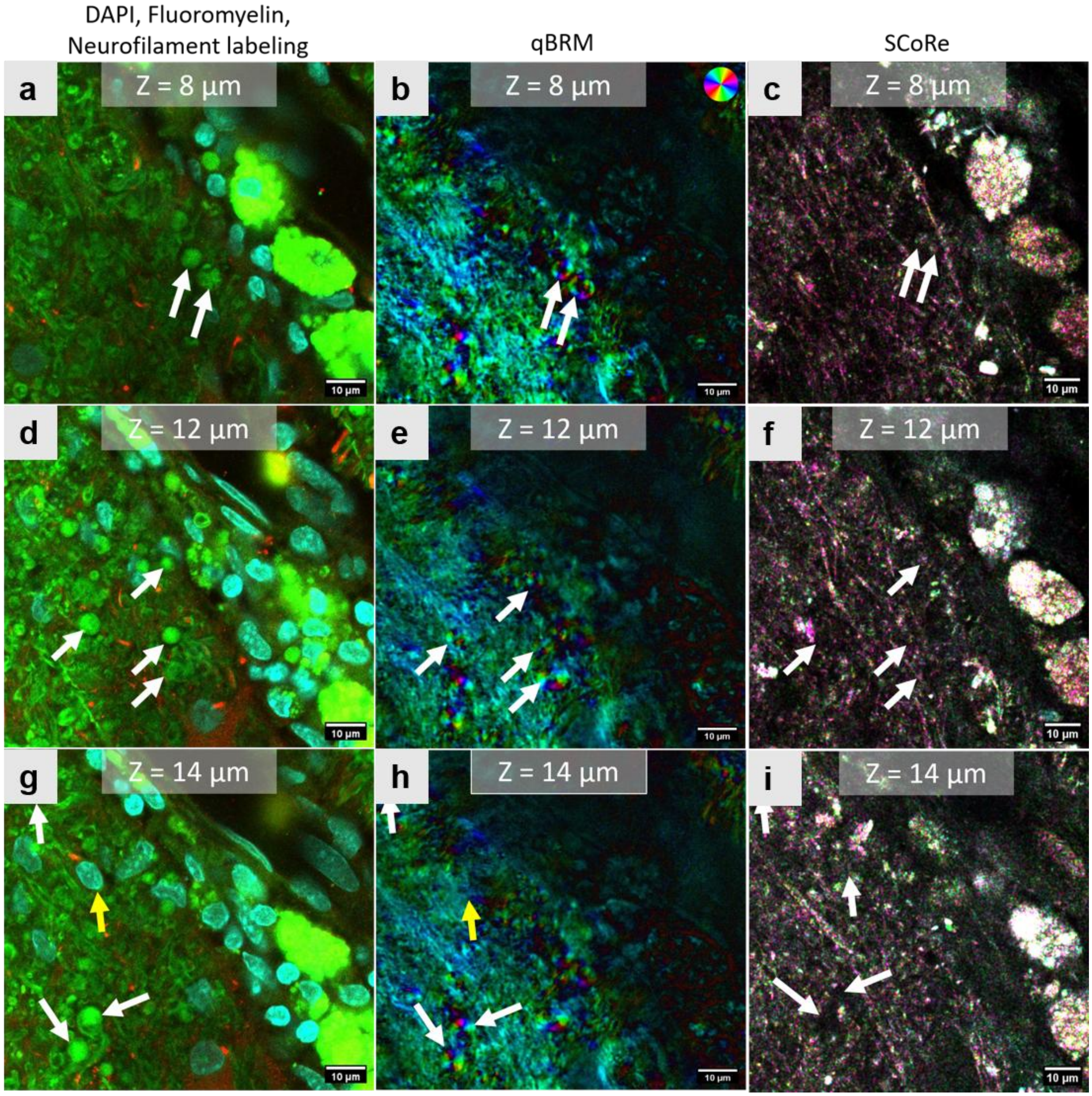

Figure S3: Comparison of vesiculated myelin as imaged by BRM, fluorescent labeling (DAPI – Cyan; Fluoromyelin (green) and Neurofilament (red)) and SCoRe imaging within the perilesional gray matter of a rhesus monkey with an induced cortical injury (Fig. 1a). White arrows indicate regions of vesiculated myelin across different focal planes (Z = 8, 12, 14 um), which are readily identifiable in both BRM (d) and IHC (e) due to their disrupted structure and retained myelin staining. These same features are not easily visualized in SCoRe images (f), likely due to the loss of reflectance in vesiculated myelin structures. Yellow arrows indicate prominent blood vessels that serve as anatomical landmarks across modalities.

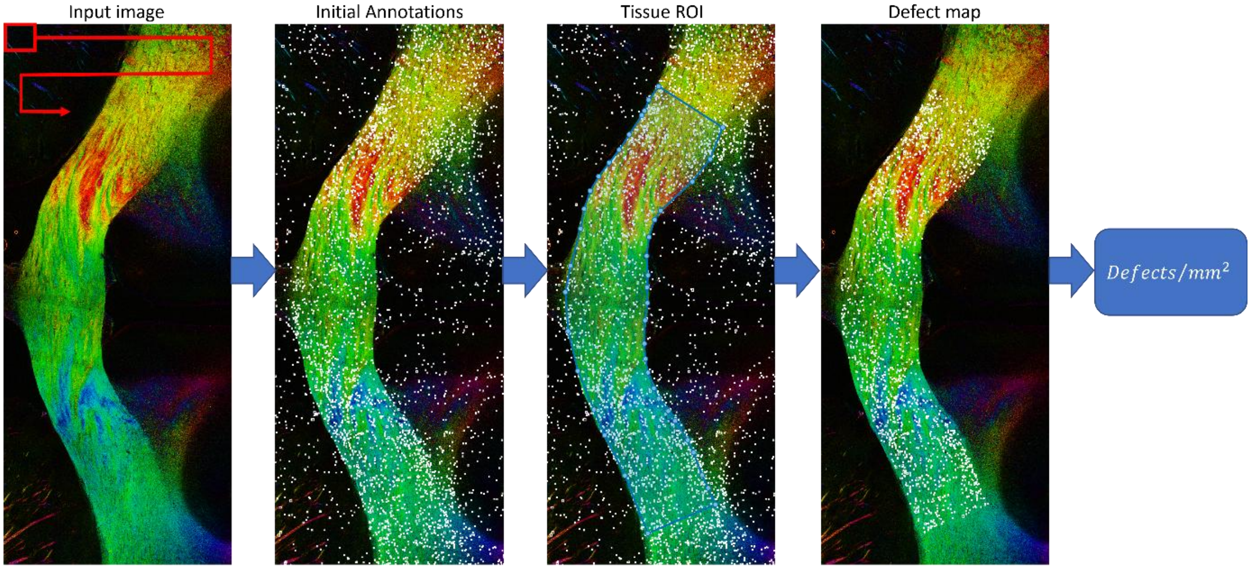

Figure S4: Image analysis pipeline for deep learning-based object detection of myelin debris in qBRM images of the corpus callosum. A high-resolution, tiled qBRM image of the corpus callosum (~35,000 × 15,000 pixels) is processed using the trained deep learning network implemented with Slicing Aided Hyper Inference (SAHI) and Test-Time Augmentation (TTA) to improve performance and generalization. Initial bounding box predictions are generated by applying a sliding window with 50% overlap across the entire image. A manually drawn tissue mask is applied to define the region of interest (ROI), with the superior and inferior boundaries determined visually and the lateralmost boundary set by a visible branch of the choroid plexus, allowing for exclusion of predictions outside the corpus callosum. The resulting defect map reveals the spatial distribution of myelin pathology, which can be further analyzed to generate a frequency heatmap or reduced to a single quantitative value expressed as defects/mm².

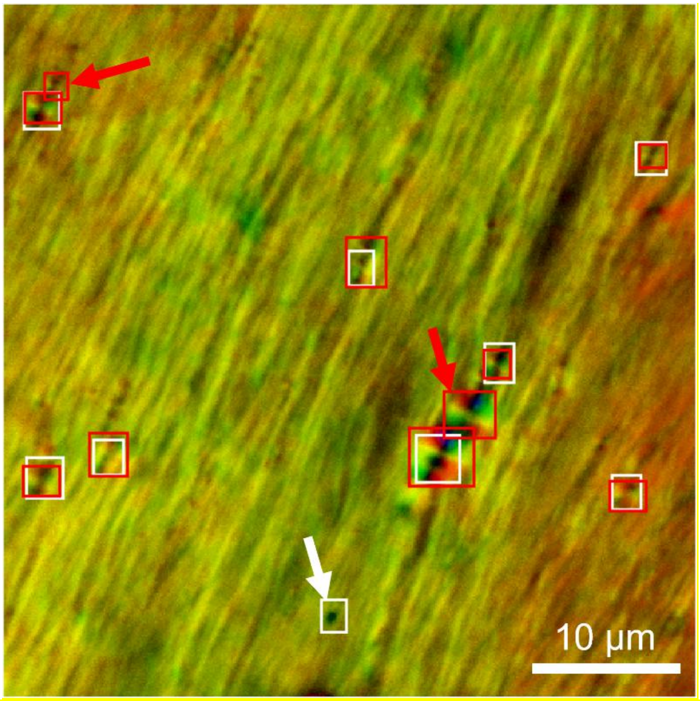

Figure S5: Representative BRM image of common errors for network annotation of myelin debris in the corpus callosum. Red boxes denote manually annotated ground-truth defects; white boxes denote network-predicted defects. Overlapping boxes indicate true positives where manual and network annotations agree. White arrows point to false positives structures which typically occur due to oblique or transverse axon segments or debris on the microscope slide that the network labels as myelin debris. Red arrows highlight false negatives, where the network fails to detect debris. False negatives typically occur in regions with tightly clustered myelin debris or small defects (can also occur due to tissue folding leading to blurry regions).

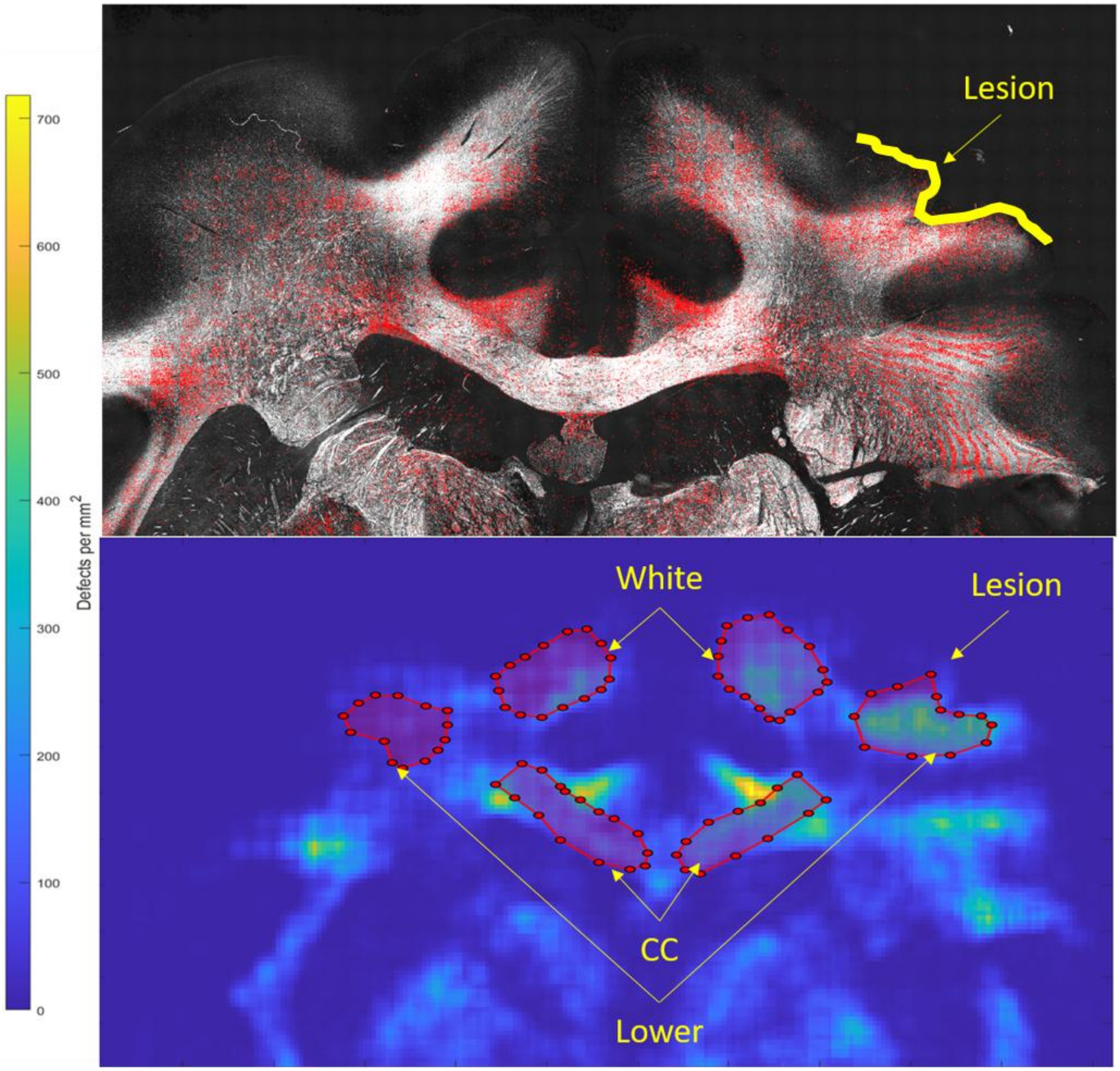

Figure S6: Whole-brain myelin debris analysis using the trained object detection network. (Top) Annotated qBRM image of a whole-brain section, where myelin debris detected by the deep learning network is marked in red. (Bottom). Heatmap representation of the relative density of myelin debris across the brain, generated using a sliding window approach. A 10X qBRM image tile was acquired across the entire brain and processed through the analysis pipeline described in Fig. S4, but applied to whole-brain sections. The trained network was developed and validated exclusively on the corpus callosum (CC) and, as a result, detects several false-positive (FP) structures across other brain regions, including oblique and transverse axons. However, assuming that these structures contribute consistently across the same brain regions, comparisons can still be made between hemispheres. To assess the relative distribution of myelin debris, the density of detected objects was calculated for both lesional side (X_L) and the contralateral side (X_C), and their ratio was computed. Representative results from this analysis show increased myelin debris density in the lesion hemisphere compared to the contralateral hemisphere across multiple brain regions: $\frac{White_L}{White_C} = 1.685$, $\frac{Gray_L}{Gray_C} = 5.103$, $\frac{CC_L}{CC_C} = 1.505$ respectively. These results demonstrate the potential of using this method to quantify myelin damage distribution across whole-brain sections. Further testing and refinement of the network, including higher-resolution imaging, will be necessary to improve its accuracy for whole-brain analysis.
