# Supplementary figures and images for: Birefringence microscopy enables rapid, label-free quantification of myelin debris following induced cortical injury
Source: Neurophotonics. 2025 Oct 28;12(4):045006. doi: 10.1117/1.NPh.12.4.045006 (PMC12576696; doi:10.1117/1.NPh.12.4.045006)

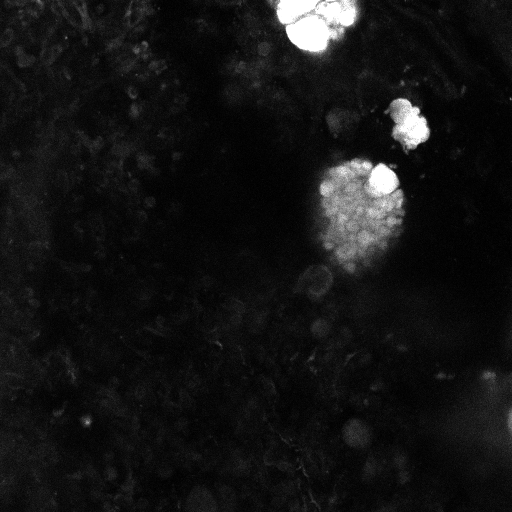

Supplement: Supplementary file 2 [file NPh_012_045006_SD002.tif]
